# Psychological support in general population during the COVID-19 lockdown in France: Needs and access

**DOI:** 10.1371/journal.pone.0251707

**Published:** 2021-05-20

**Authors:** Caroline Alleaume, Pierre Verger, Patrick Peretti-Watel, the COCONEL Group

**Affiliations:** 1 Southeastern Health Regional Observatory (ORS Paca), Marseille, France; 2 Aix Marseille Université, IRD, AP-HM, SSA, VITROME, Marseille, France; Georgia Southern University, UNITED STATES

## Abstract

**Introduction:**

With France one of the European countries most strongly affected by COVID-19 in the spring of 2020, French authorities imposed a nationwide lockdown for 8 weeks (March 17–May 10). This study explored the perception of the adult population about the need for—and access to—psychological support from health care professionals (HCP) in response to concerns about the psychological needs during lockdown.

**Material and method:**

This online cross-sectional survey of a representative sample of the adult general population of mainland France (N = 2,003) took place during the last four days of the French lockdown (May 7–10, 2020).

**Results:**

One in eight respondents (12.2%) perceived a need for psychological support from an HCP during the lockdown; most had symptoms of depression and/or anxiety of at least moderate intensity. Only a third (29.8%, 3.6% of the entire sample) actually obtained this support. Factors associated with this perceived need included: age under 35, economic difficulties due to lockdown, pre-lockdown use of psychological support, infection with COVID-19, serious worries about becoming infected, and heavy media use to obtain information about the disease. Among those who perceived a need for psychological support, the elderly were the most likely not to consult an HCP. People aged 35–64, those with high income, and those seriously worried about developing COVID-19 were the most likely to forgo seeking access to care because of their fear of infection by the coronavirus-2019.

**Conclusion:**

The perceived need for psychological support from an HCP and access to it appeared to be strongly associated with COVID-19 exposure factor. More research about this association is needed to improve the health authorities’ understanding of the population’s psychological needs in this situation and to enhance HCPs’ abilities to meet them. In particular, further research of its specific impact on youth is necessary.

## Introduction

The spread of the coronavirus disease-2019 (COVID-19) around the world starting in early 2020 led many countries to impose strict disease-control measures, such as locking down their entire population for several weeks to limit the transmission of the virus. More than 3 billion people worldwide were suddenly confined to their homes and forced to deal with major changes in their daily lives, concerning their work, their children’s education and daycare, and much more. In Europe, after Italy, Spain, and the Czech Republic, France decreed a national lockdown that began March 17 and finally ended on May 11, 2020. People whose jobs were deemed essential were allowed out of their homes to go to work. Otherwise, they could go outside for necessary shopping, for physical activity (within one kilometer of their home), and to help vulnerable populations. At the same time, the French public health authorities reported the number of infections and deaths due to COVID-19 daily and issued recommendations for preventing infection. While these measures were unprecedented in France, more local quarantines had occurred previously in various places around the world. A recent study reviewed these cases highlighting the psychological impact of quarantine [1]. Strong psychological distress has been identified among staff, hospital employees, and members of the general population who were quarantined. Lockdowns, fears about their own health or that of their families, lack of information, loss of usual routine, reduced social and physical contact with others, and financial loss were cited as causes of psychological disorders. Experts have thus warned about the potential psychological impact of the COVID-19-related lockdown measure [2–5] and the related-media coverage [6], warnings confirmed by recent studies [7–10]. Moreover, two Chinese studies suggest an association between media exposure to information about the COVID-19 epidemic and psychological distress in the general population [11, 12], thus confirming an association already shown in the literature [13, 14]. More generally, other studies have demonstrated, however, that some people with symptoms of mental disorders did not have psychological follow-up or did so only after a time lag, due either to lack of diagnosis or the individual’s refusal or both [15, 16]. For example, elderly and women presented more positive help-seeking attitude and reported more favorable intentions to seek help. To our knowledge, no published studies assess the wishes of the general population for psychological support from a health care professional (HCP) when facing a pandemic-induced lockdown.

In France, psychological support from a health care professional is not covered by the social security system unlike the majority of drug to treat mental health disorders (such as antidepressant). Thus, there is no registry for the use of psychological support from a HCP. Some study investigated the prevalence of social support use in general population outside the COVID-19 context and found a prevalence around 10% (7% in general population of 4 regions [17] and 12% among population aged from 20 to 60 [18]).

This study aimed to extend knowledge about the perceived need for psychological support among the general population locked down during a pandemic. It was motivated mainly by the following questions: How many people received psychological support from an HCP during the national lockdown? Who needed it? Did they receive the psychological support needed? And if not, why not? The main objective of this study was therefore to identify the sociodemographic profiles of people who perceived a need for psychological support and to explore the potential impact of lockdown and COVID-19 exposure factors. A second objective was to identify the main characteristics associated with access to support from an HCP.

## Materials and methods

### Design and sample

This cross-sectional online survey took place during the last four days of the lockdown in France, on May 7–10, 2020, among a representative sample of the adult population of mainland France (n = 2,003). A sample was randomly selected from an online research panel of more than 750,000 nationally representative households, who had already given their consent to be registered in this research panel, developed, and maintained by IFOP (Paris, France), a survey research firm. A quota sampling method was applied to obtain a sample of 2,000 respondents, representative of the adult general population in France for age, gender, occupation, and rural/urban residence. To limit selection bias, panelists with low response rates (i.e., panelists aged between 18–24 years old, workers, and intermediate occupations) were oversampled relative to others. Finally, 2,003 panelists responded to the survey during the study period. Respondents gave their consent to participate to our survey by clicking on the participation link on the invitation mail. They were not compensated to participate. The Ethics review board of the University Hospital Institute Méditerranée Infection approved the study design (#2020–018).

### Data collected

#### Main indicators

Our questionnaire included the following items: “Have you had psychological support from a health care professional during the lockdown? Yes/No”; If “No”, “If you did not receive such support, did you feel the need for such support? Yes/No”; and if “Yes” to this latter, “If you felt the need for support, and did not receive it, for what reason(s)?” Several non-exclusive reasons were then offered to those who reported both perceived need and lack of psychological support: failure to obtain an appointment with a physician, avoidance of potential exposure to COVID-19 during consultation, economic reasons for forgoing a medical consultation, and domestic constraints. The indicator of perceived need for psychological support was then constructed by combining the people who received this support during the lockdown and those who did not but reported that they felt the need.

#### Other indicators

To characterize this need, we used the Patient Health Questionnaire-9 (PHQ-9, 9 items) and the General Anxiety Disorder-7 (GAD-7, 7 items) to screen respectively for depressive symptoms and a generalized anxiety disorder during the previous two weeks [19, 20]. A cut-off point of 10 was applied for the PHQ-9 to identify individuals with depressive symptoms of at least moderate intensity, and a cut-off point of 10 on the GAD-7 to define anxiety of at least moderate intensity. The choice of these cut-offs was motivated by repeated expert recommendations that respondents with scores above them should be referred to an HCP [19–21]. Conversely, respondents with no anxiety or depressive disorders were identified as those with a PHQ-9 score less than 5 and a GAD-7 score of 5 or less.

Other questions inquired about socioeconomic and demographic characteristics, such as gender, age, rural/urban residence, education level, occupational situation before and during the lockdown, and financial situation. To describe the conditions of the lockdown, respondents reported their housing (overcrowded housing defined as a living area under 18 sq.m. per person or under 25 sq.m. for a single person) and its impact on their financial situation. Participants were also asked if they had been diagnosed with COVID-19, if any friends or relatives had been diagnosed and, if so, admitted to an intensive care unit, and the extent to which they worried about becoming infected (scored on a 10-item scale from 0, not worried at all, to 10, very seriously worried). The questionnaire also addressed their media consumption for information about COVID-19 during the lockdown, asking respondents how much time per day (less than 30 minutes, 30 minutes to 1 hour, 1–2 hours, 2–3, 3–4, 4–5, >5 hours) they had spent looking for information about COVID-19 from five different media sources (television, radio, newspaper, online websites, and social media) in the past week. As answers to these five items were positively correlated (Cronbach’s alpha: 0.80) we summed them to obtain a score, and we used its fourth quartile as an indicator of high media exposure (corresponding to at least 4 hours daily looking for information about COVID-19). Finally, the questionnaire also included information about their history of psychological support, to identify vulnerable population: respondents reported if they had a psychological consultation with an HCP during the past year (before the lockdown).

### Statistical analysis

We first described perceived need for psychological support and access to it, including reasons for non-utilization, using PHQ-9 and GAD-7 scores to explore the association between this perceived need and potential depression and anxiety disorders. Next, we identified variables associated with the perceived need for psychological support by bivariate analysis with Chi-square tests to compare the sociodemographic characteristics of people who did and did not perceive the need for psychological support. The potential impact of the lockdown on their individual situation and their direct and indirect COVID-19 exposure were also compared between these two groups. These associations were confirmed by a multiple logistic regression. We finally performed two other multiple logistic models to study factors associated with the lack of psychological support, respectively considering: 1/ factors associated with having received psychological support among respondents who reported a need for it, and 2/ factors associated with forgoing psychological support for COVID-19-related reason, among those who did not receive it. The tables present only the significant variables, except for gender and age; both of these variables were forced into each model to enable comparison with the literature. In addition, systematic adjustments were made for history of psychological consultation to control for previous need. Variables significant at *P* < 0.20 in the univariate analyses were eligible for the multivariate models. The final models were selected by a procedure based on the statistical significance of the covariate (probability threshold = 5%). All statistical analyses were performed with SAS version 9.4 (SAS Institute, Cary, NC, USA).

## Results

As Table 1 shows, 12.2% of participants reported that they needed psychological support from an HCP during the lockdown: 3.6% received this support and 8.6% did not. This Table also reports the mental health indicators according to perceived need for this support and shows that symptom intensity indicators were significantly higher for people who perceived their need for support than for those who did not. Overall, the prevalence of anxiety symptoms of at least moderate intensity in the study population was 21.2%, and the prevalence of depression symptoms of at least moderate intensity 22.9%. Respondents who reported receiving psychological support during the lockdown had the highest prevalence of at least moderate intensity depressive (80.2%) or anxiety (60%) symptoms. The corresponding prevalence was somewhat lower among people who perceived the need for psychological support but did not receive it, at 61.0% for depressive symptoms and 55.5% for anxiety. Conversely, people who reported they did not need such support had these psychological disorders significantly less often (only one out of six), while more than half (52.1%) had neither anxiety nor depressive disorders.

**Table 1:** Perceived need for psychological support from a healthcare professional in the general population in France and mental health indicators (COCONEL 2020, May 7–10, N = 2,003).

	Perceived need for psychological support from healthcare professional			All
	No	Yes, received some support	Yes, did not receive support	
All (row %)	87.8	3.6	8.6	100
Mean GAD-7 score^###^ (sd)	4.5 (5.0)	11.1 (6.2)	10.4 (5.9)	5.3 (5.5)
% respondents with anxiety of at least moderate intensity (GAD-7 ≥10)***	16.2	60.0	55.5	21.2
Mean PHQ-9 score^###^ (sd)	4.6 (5.1)	14.6 (6.4)	11.6 (6.9)	5.6 (5.9)
% with depression of at least moderate intensity (PHQ- 9≥10)***	16.8	80.2	61.0	22.9
% without any anxiety or depressive disorders (PHQ-9 <5 and GAD-7 <5)***	52.1	6.2	9.6	46.7

T-test

^#^*P* < 5%

^##^ *P* < 1%

^###^ *P* < 0.1% (Mean for the group “No need” as reference for comparison of means).

Chi-Square test

* *P* < 5%

** *P* < 1%

*** *P* < 0.1%.

Population: respondents for the May 7–10 wave of the COCONEL survey (N = 2,003).

**Fig 1:**
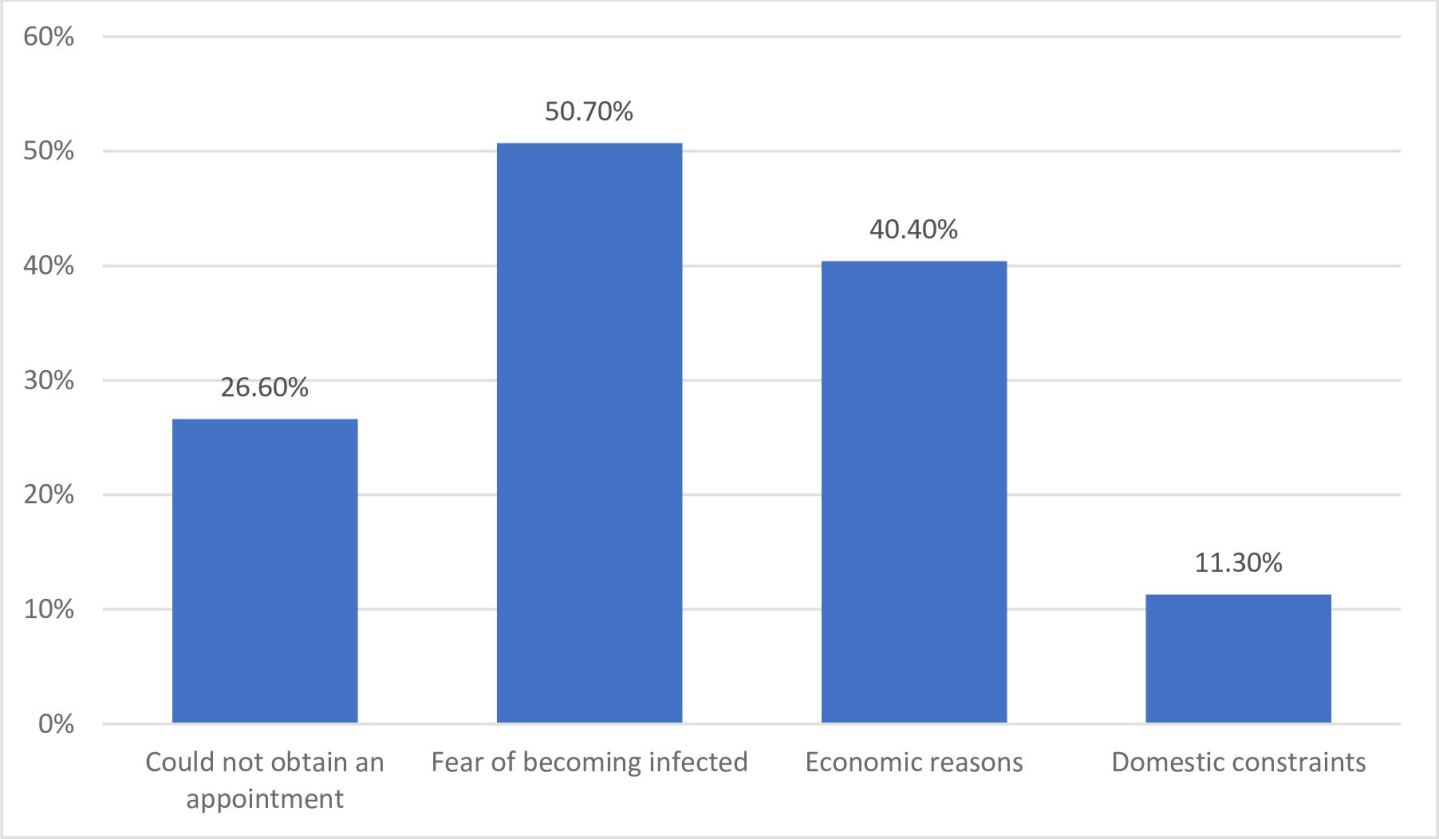
Reasons for lack of psychological support (COCONEL 2020, May 7–10, N = 245). Note: The items were not exclusive (a respondent could choose several reasons). Population: respondents for the COCONEL survey May 7–10 wave who perceived a need for psychological support from a health professional (N = 245).

Fig 1 illustrates the reasons for lack of psychological support from an HCP among those who felt they needed but did not receive it: half of them (50.7%, 4.4% of the entire sample) chose to forgo care because they did not want to take the risk of COVID-19 infection to protect themselves or their families. Moreover, 40.4% (3.5% of the entire sample) chose not to consult an HCP for economic reasons. The people in this group more frequently had a low household income level (43.1% compared with only 20.8% among those receiving this support or forgoing it for other reasons, *P*<0.001). The other reasons were chosen less frequently: 10.2% reported that they could not obtain an appointment (2.3% of the entire sample), and 7.9% mentioned domestic constraints (1.0% of the entire sample). Finally, 7.7% reported still other reasons, including their own unwillingness to have such support, having no idea how to proceed, or being on lockdown far away from their physician. But these responses were too rare (only 0.7% of the whole sample) and too diverse to be pooled into a new category.

Table 2 summarizes the factors significantly associated with a perceived need for psychological support from an HCP after adjustment for different characteristics. One respondent in 10 perceived this need among those who had not consulted for psychological support the last year. Nonetheless, history of psychological consultations in the 12 months before lockdown was the factor most strongly associated with perceived need for psychological support during the lockdown. In addition, age, the rural/urban character of the area of residence, and economic difficulties during the lockdown were significantly associated with this need: the youngest (aged 18–35) were more likely to feel a need for psychological support than the oldest. People with financial difficulties due to lockdown were also more likely to report it. A diagnosis of COVID-19 was also strongly associated with this need. Finally, serious worry (score ≥8) was also positively associated with perceived need for psychological support as was high use of media for information about COVID-19. All of the aORs for these COVID-19-related variables were of the same order of magnitude.

**Table 2:** Variables associated with perceived need for psychological support. Multiple logistic regression (COCONEL 2020, May 7–10, N = 1,996).

		Perceived need for psychological support	
		*Row %*	Model 1. With no symptom disorders
			*Adjusted OR*
			*[95% CI]*
**Gender**			
	Men (n = 949)	11.7	0.96 [0.71–1.29]
	Women (n = 1,047)	12.6	-1-
**Age**		***	
	< 35 y.o (n = 513)	17.0	1.57 [1.11–2.21]*
	35–64 y.o (n = 992)	10.1	-1-
	> 64 y.o (n = 491)	11.4	1.32 [0.91–1.92]
**Area of residence**		***	
	Urban (n = 1,550)	13.7	-1-
	Rural (n = 446)	7.0	0.48 [0.32–0.73]***
**Perceived financial situation**		***	
	Very difficult/difficult due to the lockdown (n = 423)	20.0	2.10 [1.46–3.00]***
	Very difficult/difficult not due to the lockdown (n = 603)	12.7	1.36 [0.96–1.92]
	No: Comfortable/very comfortable (n = 970)	8.5	-1-
**Locked down in overcrowded housing**		***	
	Yes (n = 176)	21.0	1.56 [0.99–2.47]
	No (n = 1,820)	11.3	-1-
**Consulted for psychological issues in the 12 months before lockdown**		***	
	Yes (n = 228)	36.2	4.90 [3.48–6.88]***
	No (n = 1,768)	9.1	-1-
**Diagnosed with COVID-19**		***	
	Yes (n = 53)	38.8	2.76 [1.42–5.38]**
	No (n = 1,943)	11.5	-1-
**Serious worry about being infected by the coronavirus (score≥8)**		***	
	Yes (n = 629)	17.2	1.76 [1.30–2.37]***
	No (n = 1,367)	9.9	-1-
**Media consumption per day for information about COVID-19**		***	
	4 h or more (n = 486)	20.2	1.72 [1.26–2.33]***
	Less than 4 h (n = 1,510)	9.6	-1-

Chi-Square test

*P < 5%

** *P* < 1%

*** *P* < 0.1%.

Covariates significantly associated with the perceived need for psychological support in bivariate analysis and not significant in the multiple regression: occupational situation before lockdown, and friends or relatives in an intensive care unit.

Population: respondents for the COCONEL survey May 7–10 wave with no missing values (n = 1,996).

Table 3 presents, among the respondents who perceived a need for psychological support from an HCP, the factors associated with receiving this support in Model 2 and with not receiving it (by forgoing/deciding not to) because of fear to get infected in Model 3. First, we see that 81.9% of the individuals diagnosed with COVID-19 received the support they needed compared with only 25.0% of those not diagnosed. After adjustment, an age of 65 or older was negatively associated with receiving psychological support, compared with a younger age, regardless of perceived need (Model 2). As in Model 1, which was presented in Table 2 and concerned perceived need, a history of psychological consultation (before the lockdown) was strongly and positively associated with receiving this support during this period. Finally, a COVID-19 diagnosis was the factor most strongly associated with reporting psychological support from an HCP.

**Table 3:** Factors associated with lack of psychological support (Model 2, N = 245) and especially with forgoing support for reasons due to COVID-19 (Model 3, N = 171) (COCONEL 2020, May 7–10).

		Model 2. Received psychological support		Model 3. Chose to forgo psychological support due to COVID-19	
		Row %	Adjusted OR [95% CI]	Row %	Adjusted OR [95% CI]
**All**		70.2	-	51.0	-
**Gender**		**			
	Men	38.3	1.51 [0.79–2.87]	50.3	0.84 [0.43–1.66]
	Women	22.6	-1-	51.4	-
**Age**		**		*	
	< 35 y.o	42.6	1.71 [0.85–3.45]	41.0	0.48 [0.22–1.06]
	35–64 y.o	26.7	-1-	62.1	-1-
	> 64 y.o	15.6	0.31 [0.12–0.83]*	44.2	0.35 [0.15–0.78]*
**Household income level**				**	
	Low	-	-	33.2	0.54 [0.24–1.24]
	Middle	-	-	50.5	-1-
	High	-	-	71.1	2.73 [1.18; 6.30]*
**Had consulted for psychological issue in the last 12 months**		***			
	Yes	48.0	4.12 [2.13–7.98]***	-	-
	No	20.3	-1-	-	-
**Diagnosed with COVID-19**		***			
	Yes	81.9	19.23 [5.12–72.29]***		-
	No	25.0	-1-		-
**Serious worry about infection by coronavirus-2019 (score≥8)**				**	
	Yes	-	-	64.6	2.45 [1.25–4.79]**
	No	-	-	39.5	-1-

Chi-Square test

*P < 5%

** *P* < 1%

*** *P* < 0.1%.

Population: respondents for the COCONEL survey May 7–10 wave who reported a need for psychological support during the lockdown (n = 245) in Model 2, and restricted to those who did not receive any psychological support during the lockdown in Model 3 and had no missing values (n = 171).

In Model 3 (Table 3), after adjustment for serious worry about becoming infected by the coronavirus, which was strongly associated with forgoing psychological support due to COVID-19 fear, people the most likely to forgo this support for this reason were those aged between 35 and 64 years and those with the highest level of income.

## Discussion

### Main results

This study shows that in the general French population, 22.9% reported symptoms of depression for at least mild intensity, and 21% symptoms of anxiety for at least mild intensity during the lockdown. Despite that, only 12.2% of the study population reported a need for psychological support from a HCP during this period, and among those, 70.2% did not receive it; people who did receive it were more likely to have more severe symptoms. In addition, this perceived need for psychological support was strongly associated with COVID-19-related factors, ranging from confirmed diagnosis to extensive consumption of media about the pandemic and its consequences, or with specific concrete difficulties during the lockdown. Youngest were more likely than their elders to report this need. Most respondents who did not receive psychological while they would like to, declared either because they chose to forgo consulting an HCP for fear of infection or financial reasons, or because they could not obtain an appointment.

### Strengths & limitations of the study

The survey conducted during the last four days of the lockdown allowed us to explore the perceived need among the general population of French adults for psychological support from an HCP, in the specific context of lockdown. Before discussing the findings, some limitations must be considered. Because lockdown has obviously affected data collection activities, online surveys are an effective way to administer questionnaires, but one that may involve some bias. The risk of missing an important segment of the French population is nonetheless limited, given that that 89% of French households have Internet access (estimation from 2018 [22]). In addition, the survey sample was generated by random sampling methods and stratified to be representative of the French population for gender, age, occupation, rural vs. urban character of residence, and region. Moreover, to limit potential selection bias, the theme of the survey was not mentioned in the invitation email. Furthermore, as the survey was conducted in the general population, the proportion of people who perceived they needed psychological support from an HCP was small (n = 245), which limited the study’s statistical power to analyze the factors associated with access to this support. Another limitation is that the low COVID-19 test positivity rate in France (around 3.6% according to the health authorities [23]) prevents adjustment for other characteristics to further investigate the association between a COVID-19 diagnosis and receiving a psychological consultation during the lockdown. In addition, the survey we used was self-administered and thus did not included any clinical diagnosis; no conclusion could be taken regarding the actual mental state of people interviewed. Depression and anxiety symptoms should therefore be clinically verified [24]. Finally, this survey was carried out in the general population and thus did not study perceived need for the most vulnerable population, that is, people in institutions such as retirement homes, or people living with a chronic disease [25].

### Most people perceiving a need for psychological support had depressive and/or anxiety symptoms

Most of the respondents, who reported they felt they needed psychological support showed depressive and/or anxiety symptoms of at least moderate intensity on validated questionnaires and, according to guidelines, should have been referred for professional help [19–21]. The prevalence of perceived need for psychological support (12.2%) in this study was lower than the prevalence of symptoms of depression (22.9%) and anxiety (21.2%), measured respectively by the PHQ-9 and GAD-7 instruments. The literature has already showed that not all people with psychological disorders feel the need for support from an HCP; this need depends on their social provision, attitude towards psychological help, and cultural or environmental influence [15, 16, 22, 23]. Conversely, not all people who felt the need for psychological support had depressive or anxiety symptoms. Therefore, the perceived need for psychological support must be considered as a full-fledged indicator to enable a better understanding of individual needs and to help public health authorities to meet them. Therefore, we did not include these variables about symptom indicators in the models to explain this perceived need. We also can assume that these anxiety and depressive disorders scales do not have optimal efficiency in the specific context of lockdown to identify psychological needs in general population but extensive studies are necessary to support this hypothesis.

### Same perceived needs for men and women but different access

The absence of an association between gender and perceived need for psychological support in our study is inconsistent with the literature, which has most often found that women are more likely than men to report both symptoms of psychological disorders [26–29] and a need for psychological support [15, 16]. This result calls into question the specific impact of the pandemic context and national lockdown. Men may have suffered more than women from the consequences of the lockdown on their work because the former work more frequently in temporary positions than the latter [30]. Given that men also appear to consider work a value essential for their personal happiness more frequently than women do [31], they may have been more psychologically affected by the negative impact of the crises on their occupational status. Another potential explanation may be that, generally, men spend more time than women outside the home for occupational activities, hobbies, and entertainment [32–35]. Being restrained to their home may be more stressful for men than women. These specific impacts of this lockdown may help to explain our result.

On the other hand, we found that, among the respondents who perceived a need for psychological support, men were significantly more likely to receive it than women (22.6% of women compared with 38.3% of men, *P*<0.01). This association is no longer statistically significant after adjustment for COVID-19 diagnosis in the multivariate analysis, probably due to the lack of statistical power when adjusting for this covariate with few people and very strong correlation with access to psychological support. When adjusting for all the covariates in Table 3 except COVID-19 diagnosis, the automatic procedure selects gender as significant.

### The youngest group was more likely to feel a need for psychological support during the lockdown

Age was an important factor associated with both the need for psychological support and access to it. Young people, that is, those 18 to 34 years, were more likely to report need for psychological support than others. Among people who needed help, the oldest were most likely to forgo it, especially for COVID-19-related reasons. These findings are consistent with the international literature, which has shown that psychological disorders during the COVID-19 pandemic have been more frequent among young adults in Italy [34], China [35], and France [36]. Young people may be more vulnerable than the rest of the population during a pandemic involving lockdown measures for two reasons. First, isolation is a sudden rupture of their lifestyle–they usually spend much more time outside the home and have a fuller social life than older people [33]. Second, students may be particularly affected by the impact of lockdown on their academic course [37, 38]. For them, this social climate could create insecurity about their professional future. Conversely, the oldest respondents were especially affected by forgoing to psychological support but not because of their fear of becoming infected with the coronavirus. They most often reported that they had no access to psychological support because they could not obtain appointments for it, possibly because of the lack of means and equipment for video consultations and/or the difficulty of access (home care services interrupted during the lockdown, impossibility of being accompanied etc.). Finally, given that people aged 35–64 were not the most worried about being personally infected by the coronavirus, we assumed that their reason for being more likely to forgo psychological support was to avoid transmitting the infection to their elders.

### Heavy media consumption and the perceived need for psychological support

Another result is the positive association between the consumption of media information about COVID-19 and the perceived need for psychological support, even after adjustment for socioeconomics, lockdown situation, history of psychological consultations, and other COVID-19 exposure covariates. This is consistent with two recent Chinese studies that observed a nearly linear positive relation between media exposure to information about COVID-19 and such psychological distress as anxiety and depression [11, 12]. In addition, Olagoke et al. [39] highlighted an indirect relation between exposure to COVID-19 news and depressive symptoms. Media consumption seems to strongly affect perceived vulnerability, a determinant of depressive disorders. In comparison, other indicators about COVID-19 exposure, including diagnosis and serious worry about becoming infected did not have a significantly greater effect on the need for psychological support from a health professional. This finding supports the recommendation made by Olagoke et al. [39] that public health professionals should work with the media to provide more psychological support as teletherapy, and more mental health resource content to their followers in pandemic situations.

## Conclusion

In conclusion, public health authorities should pay more attention to individual needs for psychological support to adapt their guidelines according to the specific context of a lockdown during a pandemic. Experts have already warned that mental health services must be prepared to face an increased number of patients after lockdown [40]. In addition, this study shows that healthcare professionals must be ready to deal with new needs of a specific population, younger and more masculine. Health authorities have recommended mental-health follow up to diagnose anxiety or depression in patients after discharge from intensive care units [41], and this guideline appears to have been followed even for people diagnosed with COVID-19 and able to respond to this online survey (and therefore unlikely to have spent time in an intensive care unit), since almost all of the diagnosed patients who needed psychological support had it. Efforts should be made both for people with anxiety or depressive symptoms who did not feel the need for support and for those in whom no anxiety or depressive symptoms could be identified but who still reported needing psychological support. On this point, more research is needed to set up psychological interventions appropriate to individuals’ needs and desires during outbreaks, especially for young people. Interventions set up during the lockdown to prevent risk of social isolation and loneliness (such as phone check-in calls made by municipalities or association groups, the provision of free hotlines for psychological support [42]) should be assessed. Further investigation about our finding concerning respondents who did not have any psychological support, although they perceived they needed it, is essential, especially about the reasons that those worried about coronavirus infection did not use video consultations. Virtual consultations have been recommended by experts [43, 44] to prevent problems from accruing untreated during lockdown [40, 45], a good way to limit the spread of infection it seems safer than face-to-face contact [46], and their use in general populations must therefore be assessed. Moreover, the new finding about the potentially seasonal nature of COVID-19 [47] should encourage health authorities to promote medical video consultations, including for psychotherapy and psychiatric follow up. This development of teleconsultation should be accompanied by coverage of psychotherapy by the social security system. Some experimentations are currently conducted in three region of France by the insurance organization National Health Insurance System. In addition to these long-term propositions, some interventions were identified as effective to reduce the mental health impact of an epidemic such as group-based cognitive behavioral therapy, psychological first aid, and community-based psychosocial arts program [48], and should be implemented and assessed in France to help people during a specific event as the COVID-19 pandemic.

## List of abbreviations

HCP: Health care professional
